# Sensemaking in the early stages of the COVID‐19 pandemic: A narrative exploration of polarised morality in an NHS Trust

**DOI:** 10.1111/1467-9566.13586

**Published:** 2022-11-11

**Authors:** Alice Faux‐Nightingale, Mihaela Kelemen, Simon Lilley, Caroline Stewart

**Affiliations:** ^1^ Keele University Keele UK; ^2^ Nottingham University, Jubilee Campus Nottingham UK; ^3^ University of Lincoln Lincoln UK; ^4^ Robert Jones and Agnes Hunt Orthopaedic Hospital (RJAH) Foundation Trust Gobowen UK

**Keywords:** COVID‐19, crisis, emotions, intuitions, moral judgement, NHS trust, sensemaking

## Abstract

This article presents an analysis of personal diaries kept by health‐care staff within a specialist NHS Trust in England during the initial 3 months of the COVID‐19 pandemic. It adopts a moral sensemaking perspective to explore how NHS employees mobilised and reframed ideas of right and wrong in order to make sense of unprecedented uncertainty and displacement. By focussing on how the macro and micro politics of the pandemic were played out in the organisation, the study finds that polarised moral judgements were invoked in order to justify and rationalise a broad array of associated emergent emotions, intuitions, behaviours and practices. This polarisation of moral responses could be seen as a desire to bring order out of chaos and put matters back into place following displacement. This is inevitably an ongoing, complex and variegated enterprise whose results can be as often discomforting as they can be reassuring. Indeed, while moral sensemaking was partly beneficial for staff in that it promoted a greater sense of camaraderie and support for others, it also appeared to have darker consequences in terms of staff wellbeing and the development of more impermeable social boundaries across the organisation through processes of moral ‘othering’.

## INTRODUCTION

The COVID‐19 pandemic is the most significant global health crisis in living memory (Nembhard et al., 2020) and its impact on public health policy is likely to continue to unfold for years to come. In the UK, the National Health Service (NHS) was met with increased demands for health care, and new policies and guidelines were developed to support the effective and appropriate distribution of patients and the management of care (Savulescu et al., 2020). Contrary to the usual methods of implementing organisational changes incrementally and after a degree of consultation, many of these new policies were enforced rapidly, causing disruption and uncertainty within the NHS workforce. Confronted with significant and ongoing institutional changes, NHS staff engaged in intuitive sensemaking to re‐establish a sense of normality and restore the moral order.

The article presents an analysis of diaries produced by health‐care staff during the first three months of the COVID‐19 pandemic. It proceeds with a review of the literature on sensemaking in crisis situations to foreground the moral complexity facing NHS staff. This is followed by a discussion of the research methods and data analysis. The results section sheds light on the polarisation of the moral frameworks invoked by staff to justify their actions, thus extending our understanding of the role played by intuitions and emotions in sensemaking in times of crisis. In addition to making a theoretical contribution to moral sensemaking, the article offers practical insights. Through surfacing and highlighting the polarised nature of moral sensitivities during times of crisis, the article provides an opportunity for NHS staff and managers to consider the implications of both individual and institutional sensemaking in times of crisis and how they might best be managed in the future.

### COVID‐19: A crisis requiring sensemaking

A crisis is an unexpected event that significantly threatens a former way of life (Kornberger et al., 2019), a situation where traditional values and rules no longer provide a compass for how to understand and act in the world. According to Weick et al. (2005), sensemaking is prompted by crisis situations that deviate significantly from the normal, making it impossible for individuals to rely on old perspectives and routines to process what is happening around them and engage in the customary flow of life. Sensemaking is both accomplished in the midst of crises (Weick, 1990, 1993) and used in their aftermath to explain them (Gephart, 1993; Gephart et al., 1990).

Some commentators render sensemaking as a *cognitive* process that helps individuals construct frameworks or mental models of how the environment works (Hill & Levenhagen, 1995; Starbuck & Milliken, 1988). Others argue that sensemaking is a *social* process that occurs not at an individual level but *between* people who engage in negotiating and co‐constructing inter‐subjective meaning as a basis for action. For example, Maitlis (2005, p. 21) argues that sensemaking occurs when ‘organization members interpret their environment in and through interactions with others, constructing accounts that allow them to comprehend the world and act collectively.’ Viewed through this lens, sensemaking is a social and discursive practice that relies on conversational and narrative resources (Brown, 2000; Gephart, 1997), where changes in the environment are seen to emerge primarily through shifts in the very language that facilitates their apprehension (Barrett et al., 1995; Ford & Ford, 1995). While power, emotion and socio‐materiality have all been recognised as important aspects of sensemaking (Maitlis & Christianson, 2014), the *moral* aspect of sensemaking has received relatively scant attention in the literature.

### Moral sensemaking and emotions

In the public health emergency entailed by COVID‐19, displacement for many NHS staff was immediate and impossible to ignore. The customary arena of work was replaced for some with the home, whilst for others, also experiencing relocation to the domestic sphere, working simply ceased for an extended period. Other staff found themselves either in new organisational spaces or, indeed, in more immediately familiar ones that were orchestrated and ordered in such novel ways that they too were subject to considerable estrangement. Such estranged workplaces became rapidly associated with new senses of right and wrong, evincing and reflecting new judgemental frameworks and repertoires of accusation, justification and exculpation.

Moral judgement becomes particularly salient during times of uncertainty when the established ‘order of worth’ (Boltanski & Thévenot, 2006), consisting of the higher common principles that define and prescribe appropriate forms of conduct for specific contexts, no longer provides an effective platform for the coordination of individual actions, compelling actors to justify themselves and give a moral account of their actions (Patriotta et al., 2011). To bridge between the normative principles underpinning the existing ‘order of worth’ and the complex, messy and affectively charged world of practice, individuals engage in processes of moral sensemaking that are highly emotional (Reinecke & Ansari, 2015). Studies show that emotion and intuition play a significant role in sensemaking in health‐care locales (Lützen et al., 2006; Theodosius, 2008) in both clinical and non‐clinical roles (Ward & McMurray, 2011) and that crises accentuate moral complexity and foreground the importance of moral judgement (Reinecke et al., 2017).

Moral judgement in organisations is often researched within the well‐established, rational ethical decision‐making literature, rooted in psychology. Here, moral judgement is seen as the result of moral reasoning, which is influenced by individual factors, such as cognitive moral development, age, education, employment, personal values, and integrity, as well as situational factors including both issue‐related and organisational factors (Jones, 1991, 2001, 2012; Trevino, 1986). In contrast, Haidt’s social intuitionist model (2001, 2012) suggests that moral judgement is not a cognitive achievement but an intuitive and emotional one, the result of quick and automatic evaluations in which social and cultural influences are more important than individual ones. Family (Flood‐Grady & Koenig Kellas, 2019; Medved et al., 2006), peers and the media (Whitbeck, 1999) shape the way that individuals make sense of their environments as well as their identities (Doherty & Saunders, 2013).

Thus, moral reasoning is not private and rational, nor does it necessarily precede moral judgement; on the contrary, it is often a post hoc construction that aims to justify one’s emotions, intuitions and actions in line with preferred higher common values and principles. Studies that have begun investigating the impact of the pandemic on moral reasoning have noticed how the pandemic has shifted public priorities and moral perceptions, and resulted in the moralisation of behaviours previously considered neutral, for example, social distancing (Francis & McNabb, 2021; Navajas et al., 2021); and how government communications and the media used this moral reframing of behaviours to promote compliance with public health behaviours (Francis & McNabb, 2021; Kasper et al., 2022). Haidt (2001) argues that in highly ambiguous situations that disturb the prevailing ‘order or worth’, reasoning processes are incapable of offering immediate or predictable outcomes; as such, the intuitive process remains the default practice to handle moral judgements in a rapid and holistic way.

Personal narratives from NHS staff working in an English Trust highlight the centrality of their moral intuitions and emotions in how they made sense of organisational changes, new work routines and the lifestyle choices that were commonplace at that time. Our study adopts a moral sensemaking perspective, drawing on principles from Haidt’s social intuitionist model and Boltanski and Thévenot’s ‘order of worth’ concept, to explore how NHS employees’ mobilisation, and reframing of ideas of right and wrong, and justification of their actions were affected by the unprecedented uncertainty of the initial three months of the COVID‐19 pandemic. We offer insights into how actors questioned salient ‘orders of worth’, which were seen incapable of providing social structure and meaning, triggering the need for localised context‐sensitive moral sensemaking. We consider how the sensemaking process became morally polarised, acting as repair work for individual employees at all organisational levels who invoked moral judgements to justify and rationalise a broad array of associated emergent emotions and intuitions. The study responds to Christianson and Barton's (2020) call for more empirical studies of sensemaking in pandemic times by exploring the moral sensemaking processes by which the pandemic was understood, felt, negotiated and translated into organisational practices in a highly visible and politicised context, the National Health Service.

## RESEARCH METHODOLOGY

### The organisational setting

The NHS Trust that took part in this study is a small, highly specialised orthopaedic trust in England. During the COVID‐19 pandemic, some of the specialist services offered by the hospital were discontinued, including the routine orthopaedic surgery elements, in which the Trust specialises. The site became a COVID‐19‐free hospital and took on some new services from nearby Trusts to enable the latter to better meet the demand for COVID‐19 positive patients. While the hospital itself did not take on significant numbers of COVID‐19 positive patients, it did care for a small number of patients with the virus during the pandemic.

### Recruitment

All staff at the specialist NHS Trust were invited to take part in a diary exercise backed by the senior management. The last author of the article is a registered clinical scientist and clinical service manager who acted as gatekeeper and liaison with the Board of Directors. An initial approval to support the use of the data for research purposes came from the Trust through the clinical audit department, and a retrospective ethical approval was obtained prior to any research activity taking place. All participants were given an information sheet about the management exercise where they were informed about the potential for use of their diary entries for research and publication prior to consenting to participate.

Thirteen members of staff consented to participate, and each produced at least two diary entries: ten were women, three were men and their ages ranged between 24 and 61, see Table 1 for further details. The only key populations from which we were not able to recruit were those who shielded in the early stages of the pandemic and those who had a reduced workload.

**TABLE 1:** Participant job title prior to the COVID‐19 pandemic and during the crisis, and number of diary entries submitted during the study

Participant	Pre‐COVID‐19 role	Role during COVID‐19 crisis	Number of diary entries
002	Lab manager	Normal role	2
003	Team lead	Team lead (clinical)	5
004	Research nurse	Staff nurse	9
005	Ward clerk—Ward A	Ward clerk—Ward B	3
009	Director	Normal role	17
014	Physiotherapist	Normal role	10
015	Outpatient supervisor	Normal role and fracture clinic support	2
016	Department manager (non‐clinical)	Normal role	14
017	Business coordinator/admin team lead	Normal role with reduced hours	5
018	Appointments supervisor	Normal role with reduced hours	6
019	Manager (non‐clinical)	Normal role	14
023	Doctor	Registrar medical cover	5
026	Consultant	Normal role	6

Each participant offered a unique experience in terms of working location, department and seniority within the organisation. Significantly, eight participants experienced a relatively major change to their working roles and/or the context in which they delivered them: one moved to working at home full‐time, whilst another five divided their time between home and the hospital. Six participants had children at home who were previously in school, or who had lived away from the house but returned during the pandemic, which changed their home dynamics and caring responsibilities.

### Data collection and analysis

Data were collected through diary entries over an observational period of 3 months, with each participant being asked to report on their experiences over a 3‐week period. The diary method has a long history in health studies (Pennebaker & Seagal, 1999), education (Platzer et al., 1997), psychology (Bolger et al., 2003) and work studies (Travers, 2011) due to its ability to give voice and agency to respondents and facilitate the capture of ‘thick descriptions’ (Geertz, 1973). Symon and Cassell (1998) argue that qualitative diaries give access to lived experiences in a way that generates detail that would otherwise take hours of interviewing to elicit. Diary methods are also well placed for exploring ‘questions concerning process and change within a person over time’ and ‘given that respondents write their diary shortly after an activity occurs, they provide for the proximity and practical relevance of knowledge’(Götze et al., 2009, p. 268). Notable personal diaries capturing doctors’ COVID‐19 experiences and moral judgements in pandemic times were put in the public domain via the publication of various books (Down, 2021; Farooki, 2022; Pimenta, 2020).

We asked participants to produce a daily entry in which they recorded their thoughts about the day. Prompts were provided as part of the management exercise; these encouraged discussion about the work challenges brought about by the pandemic and included questions about organisational responses to COVID‐19 in terms of work arrangements, organisational structures, processes and communication mechanisms. However, participants were also encouraged to write freely about how they were affected by the pandemic at work and home and include anything they thought was significant about their day. Participants’ entries addressed the management prompts to varying degrees, but all included personal stories that were unrelated to the management questions. No participant produced a daily entry for the full 3 weeks; the number of entries varied across participants, ranging from 2 to 17. After one follow up email, no additional effort was made to encourage participants, as it was recognised that it was already a stressful period of working life. Diary entries were initially requested as audio entries to minimise the time demands on the participants, but several participants submitted written entries instead, due to concerns about anonymity, perceived ease of access or simply out of preference.

All audio recordings were transcribed and anonymised by medical secretaries within the Trust before being sent to the researchers for analysis. The participants were assigned numbers as pseudonyms, and any hospital‐specific roles were similarly pseudonymised. A preliminary thematic analysis of the diary entries was carried out at the end of each week, and emerging themes were used to guide the development of prompts for the subsequent week. Once the observational period had closed, a reflexive thematic analysis (Braun & Clarke, 2022) was carried out independently by each author, who subsequently compared their results. The authors identified a number of commonalities across individual coping strategies: narratives about moral tensions and difficulties in making sense of broader social and cultural influences, internal and external to the hospital were key. Following these considerations, four central themes emerged: rate of change, sensemaking, morality and emotion.

### Reflexivity

Although initially positioned as a management exercise, participants were fully aware and consented to the data also being used for research purposes prior to participating. Questions were asked as part of the management exercise, but all participants were encouraged to write freely about the highs and lows of daily life. Although the diaries were seen by some participants to be a mere personal account or to function as a therapeutic device, they were undoubtedly affected by the knowledge that the entries would be read by external figures. Indeed, some might have treated their diary entries as a means to communicate with the senior management team in the knowledge that researchers would be passing information back to the directors, albeit in anonymised form, for example,: ‘*management aren’t going to like the fact that I’ve said this*’ 016. The awareness of the use of the study as a management exercise and the potential position of the narrative as a tool to promote change in the workplace might have influenced how individuals recorded their experiences and explained why they preferred a highlights‐style narrative rather than documenting every daily occurrence. The variegated responses we received suggest that while some participants did have a senior management audience in mind, many diaries included personal reflections and intimate stories about things happening in their private lives and how these intersected with work matters. As such, the participants did not appear to feel in any way restricted or pressurised to focus solely on the management prompts, and we found that, while there was some variation in the recording style, participants’ attempts to make sense of the pandemic via diary writing were underpinned by a heightened moral orientation.

As researchers, we were acutely aware of our own sensemaking processes and the need to be reflexive and mindful of our own positionality. Having an NHS practitioner as part of the research team brought balance to the data analysis and allowed us to orchestrate participants’ voices in ways that illuminated both personal and institutional challenges. We reflected in particular on how the nature of the exercise and the context in which it was undertaken had influenced both the production of personal accounts and our own reading and interpretation of participants’ narratives. By focussing on the processes of sensemaking that occurred as part of the diary writing exercise, we were able to foreground the centrality of moral judgement in times of crisis.

## RESULTS

### Rate of change

All participants drew attention to significant changes that had arisen in their working environment in response to COVID‐19 and to the associated confusion and uncertainty triggered by constant changes of policy and practice. Many participants described how changes seemed to be enacted rapidly with little explanation or communication with those who were affected. It was clear that for many of the participants, the perceived rate of change and how management seemed to have implemented the new policies were as significant as the new policies themselves:

There’s lots and lots of change occurring on a daily basis. I know things are changing, you know new guidance comes out from PHE [Public Health England], that is great, but it doesn’t really seem to get fed out properly so that we know where we are. We are told one day this is what we are doing and then the next day you find out from somebody else ‘oh, no, you don’t do that, no you do this’, but again it just comes down to nobody being really sure what it is that we are supposed to be doing. […] It just feels like every day you come in there is yet another set of rules. I had a couple of days off last week and turned up to work on Saturday to find another A4 sheet of paper with a list of rules on it.

(003)

Staff described the physical and emotional impact of this rapid rate of change. Many participants complained about working at a higher rate than expected, which contributed to feelings of stress and burn‐out and affected the way they interacted with their jobs and with each other. Participants regularly used words such as ‘draining’, ‘relentless’ and ‘total exhaustion’ to describe their working lives, rendering the early stages of the pandemic as ‘chaotic’ and like ‘firefighting on a daily basis’. Such negative emotions were expressed in many diary entries and often used to explain existing low energy levels and moods. Participants also described how they perceived the pandemic was affecting their colleagues, with those who were responsible for offices or departments being particularly prone to reflecting on the impact on their subordinates, who were seen to be tired and struggling with the relentlessness of the situation.

The NHS actions taken in response to the pandemic and their public justifications appeared to trigger moral tension for some of the staff who took part in the study. At the heart of the tension was the dissonance between the NHS messages presented to (some of the) staff and those presented to the public: the wider public were told to remain at home to protect themselves and others from the virus, while many health‐care staff were encouraged to come to the ‘front line’ to care for the sick. This contrast in demands was similarly seen within hospital staff. Most (typically non‐clinical staff) were sent away from the hospital to avoid the risk of catching and spreading the virus, while others (typically clinical staff) were told to remain working on site or were moved to clinical roles where they were at a higher risk of catching the virus. This apparent tension between staff being asked to work on site and staff being sent to work from home provoked negative emotions such as worry and concern, as reflected in some participants’ narratives:

It didn’t take a genius to work out that as a trained nurse I was soon going to be deployed onto a clinical team, although this wasn’t confirmed. The following week I was on annual leave and the press was crazy at that point asking all nurses and doctors to return to the NHS and I felt quite worried and concerned all week, I was thinking about who am I going to be looking after, where am I going to be working.

(004)

Staff working on site wrote about the negative emotions provoked by a perceived risk that they could infect their own families and the increased worries family and friends expressed about their safety at work:

I resisted calling my mum as she is really worried about me, I felt silly to tell her that I may have been putting myself at risk, not from the patients but from the staff, so I, I haven’t actually spoken to her since then.

(004)

### Sensemaking and the polarisation of moral orientation

Participants’ sensemaking processes were apparent in those entries that recounted constant changes surrounding the work environment. Many entries featured long sections where participants explored the emotional effects of rapid organisational changes, including, in their own words, ‘rants’ (009) replete with personal feelings:

I’m not saying that we shouldn’t have rules, we know we need them…but, you know, just a little bit of an explanation would help. Whoever is writing these, just please explain to us, please don’t leave the speculation to everybody, because everybody is putting two and two together and coming up with five. People are getting angry, people are getting upset, people are feeling like it’s a personal attack. You know, where do you go from there? How do you try and put that right? How do you try and tell people, how do you pull a positive out of that, how do you kind of say, well look these are the rules and it’s just the way it is, when people are feeling stressed and they’re feeling worried and they’re feeling anxious, just one more set of rules on an already sort of twentieth set of rules, if you like, is just not really helpful. It is hard not to feel disillusioned. It is hard not to feel demoralised. You know you do your absolute best, everybody is doing their absolute best but it still never really feels like what you are doing is good enough or ever will be good enough.

(003)

In later prompts, participants were asked if their priorities and ways of thinking had changed due to the pandemic:

Does it change the way I think about things? Well I mean, yeah, it kind of, you put others first don’t you, as you think that your actions are probably, directly, going to impact on, on other people’s health, so it’s kind of, you put your own needs to the bottom of the pile.

(014)

Participants’ narratives increasingly displayed polarised moral concerns associated with COVID‐19‐related behaviour. Responses commonly presented a view of the workplace structured around moral, almost archetypal, views of ‘good’ and ‘bad’, which aligned to a large extent with the images in the media at the time and the core values of the NHS. Instances of ‘morally right’ and ‘morally wrong’ attributions reverberated throughout the diary entries, with participants expressing a need for everyone, including themselves, to do the ‘right thing’ in the pandemic, and frustration and condemnation when individuals did not.

### ‘Morally right’

Participants nearly all framed their narratives in terms of how their thinking and behaviour aligned with higher common principles, that is, the six core values of the NHS: Respect and dignity, Commitment to quality of care, Compassion, Improving lives, Working together for patients and Everyone counts:

I am happy to do whatever I can to help the Trust run as smoothly as possible and am quite happy to change my days/working pattern to suit what is required.

(018)

I don’t do anything at all for thanks, I do it because I just want to be the best I can, if you get what I mean. I kind of just want to be able to give as much support and help to my colleagues and be the best that I can be for my patients.

(003)

Other staff emphasised working harder or longer hours due to the demands of the pandemic:

I’ve been logged on since 7:30 and I’m still working at 6 and haven’t finished yet.

(016)

Narratives frequently included expressions of empathy and of staff appreciation, both as comments directed towards other members of staff and where they had been received from others. These were most prevalent in the narratives of staff who had remained working on site. Accounts featured regular occurrences of increased staff camaraderie and support, considered to have developed as a consequence of the pandemic:

I’ve certainly felt really supported by my colleagues, in a way that probably I haven’t felt at [this hospital] for many, many years, it really feels that people are actually watching each other’s backs and are there to support you, accepting when you are not having such a good day and that, that camaraderie has been really special.

(003)

There’s a really good feeling in the hospital, the camaraderie is very very good, people always stop and talk, people are very chatty, people smiling ‐ it is a very much we're all in this together type thing.

(005)

As expected, specific COVID‐19‐related behaviours and regulations were also incorporated into the narratives, with many of the participants discussing their willingness to engage with the new measures. These narratives were overwhelmingly positive as participants generally regarded the new measures as fair and necessary and were willing to comply, often from a desire to better protect the people around them:

I am keen to observe the rules which are in my opinion correct in order to prevent further spread of the virus.

(018)

### ‘Morally wrong’

The strengthened identification with morally good behaviours cast a shadow on individuals or situations where what was deemed to be the morally right choice was not apparently being made. Some participants described frustration in situations where people had changed their behaviour due to the COVID‐19 pandemic but had not internalised the NHS ethos. People who were deemed to have enacted behaviours or made decisions for their own benefit rather than for the benefit of others or who exhibited what was deemed problematic behaviour were perceived particularly negatively, and attention was drawn to these instances of conduct in the diary entries:

I think I just feel disappointed that, you know, people have used [the pandemic], it almost seems to be an excuse for behaviour, it almost seems to be, you know, that, oh everybody’s tired, everybody’s stressed, everybody’s worried, well they are, but, you know, we can’t keep saying it’s because of Covid‐19 that so and so’s behaved like that or, you know, sometimes surely Covid‐19 or no Covid‐19 we, we have to take responsibility for our actions and our behaviours.

(003)

It was interesting to see different interactions between colleagues, how, maybe because of the Covid‐19 situation and how precious and scarce operations have become, how people’s personalities per se have changed, I don’t know the word to be honest, but they (the surgeons) have become a bit more crafty, I would say, manipulative.

(023)

Many participants alluded to their expectation that certain colleagues must move towards a more morally good stance, whether in terms of emotional or behavioural responses:

I think what I’d hoped to see, or what I thought I would see, was that people would be more compassionate towards each other, more mindful, of each other. […] not looking to see how we can score points over one another and who can shout the loudest because that makes you the most important person and you know we just don’t need to be doing any of that at the moment. We need to be, we all need to be more mindful.

(003)

Interaction with COVID‐19 measures, or lack thereof, was a common focal point in narratives. Here, participants often expressed discomfort with individuals seen not to be adhering to rules as well as a desire for these staff to adhere more closely:

Social distancing, is a real issue for me in the sense that I get frustrated in two different ways really in the sense that you see people, particularly clinical staff, I just don’t get it, that they actually even lift up their masks and will stand really close together, I don’t really understand that, but the vast majority of people are doing their best to practise at the 2 metre social distancing.

(003)

Coupled with opprobrium towards others’ perceived questionable conduct was a concern about a perceived lack of equality in the ways in which policies and changes were applied to the staff. Talk about things being unfair and demands for situations to be made more equal were common in the diary entries, particularly those produced by staff who were not offered reduced hours or the opportunity to work from home. The moral undertone of the differences in workloads across the Trust is aptly captured by an administrator:

It became become very apparent to me over the last few weeks just how very dissatisfied a lot of people in the Trust are, and this‐ mainly due to them feeling umm that they are being unfairly treated compared to their colleagues. Up until today I’ve not been too bad about it but then today I think that that it’s suddenly hit me…it’s just the fact that nobody wants to feel that they’re being taken advantage of or being made a mug of. Now this isn’t just me, this is across the board, this is nurses, this is HCAs, Ward Clerks, admin, this this is constant, this is what you are hearing all the time from the people who are turning into work and working their normal hours every week.

(005)

### Moral sensemaking and emotions

Persistent focus on actions that embodied moral rectitude imbued many accounts, with some describing feeling pressure to support colleagues and patients beyond normal expectations:

I think because I’ve probably inadvertently put probably a bit more pressure on myself than I’ve maybe realised, I don’t know if pressure is the right word really. You know to kinda want to make sure that I am up to speed as much as I can be in order to help the staff, in order for them to feel less stressed and for them to feel comfortable, I’m probably not realising how much I’m actually sort of coming home, doing what I’m doing to facilitate that and the pressure I’m probably putting on myself.

([sic] 003)

With participants applying pressure to better adhere to their own seemingly enhanced moral standards, the narratives referred to feelings of guilt triggered by the perceived inability to consistently achieve this across the board. This was particularly common for staff who had children at home but had been unable to dedicate time to them due to work demands:

I do feel quite guilty that I’m‐, I’m not able to do what I think I should be doing. There’s a lot of pressure with, with the social media and on the BBC about what it’s like to be home schooling and actually from my perspective, I’m not doing anything at all, the kids are just having to muddle through themselves.

(009)

There were also examples of guilt in the working environment, particularly where participants were working reduced hours or in alternative circumstances, with participants feeling that they were not doing as much as others or enough during the stressful time:

It has worked out that I am doing two days a week. There are some feelings of guilt that I am not doing enough.

(018)

In some instances, the emotions contributing to sensemaking were raw, triggering frustration towards the organisation alongside misunderstanding and tears:

Two weeks in I had a ‘moment’. The realisation that this (the pandemic) was real, hit all of a sudden, and that service continuity rested solely on me and my health. The trigger was an email in relation to laptop and VPN access. I misread the email and was convinced I was going to have my laptop taken away. This had me in tears and ready to chew somebody’s ear. A colleague talked me down… I’ll always be grateful to him for being so kind that day.

(002)

## DISCUSSION

Stories and narratives have been shown to be useful tools for shedding light on the sensemaking processes in which people in health care engage when faced with situations of extreme uncertainty and change (Doherty & Saunders, 2013; Greenhalgh et al., 2005; Rhodes et al., 2016). This study makes three key contributions: it advances the literature on moral sensemaking in crisis times, offers rich and nuanced qualitative records of NHS staff experiences during the initial months of the COVID‐19 pandemic and provides practical implications for institutional practice and policy.

### Moral sensemaking and emotions during COVID‐19

Narratives transmit cultural values and moral norms through processes of inheritance as they are passed down through families, communities and other relevant social structures (Goodall, 2005). A significant contributor to the anxiety brought about by the COVID‐19 pandemic was a complete lack of narrative inheritance from previous generations. Health‐care staff were particularly affected as the NHS became a priority service: NHS staff were subjected to pressures from the public, including via acclaim, applause and higher expectations, but also through the uncertainty caused by multidimensional displacement in their workplace and worries and concerns from and for their families.

Staff diaries described not only the physical and policy changes experienced in the workplace but also how such sudden changes were integrated into a moral (re)orientation and the emotional impact that this had on the staff. The change was sudden, and this was regarded as being out of character for the NHS in its typical pre‐pandemic mode of operation. The early months of COVID‐19 brought manifold displacement for the participants, with ‘chaotic’ changes unlike anything they had experienced previously being enacted rapidly and with little accompanying explanatory information. Coupled with the wider social changes that were affecting the public at the time, staff experienced a set of interrelated displacements that triggered substantial shifts in sensemaking (Weick et al., 2005).

Health‐care settings already integrate moral sensemaking processes into daily practice, for example, when considering treatment options or waiting lists (Browning, 2012), though it was clear from the diary entries that individuals’ sensemaking expanded beyond work to consider wider impacts of the pandemic on their public and private lives. This may not come as a surprise, as it is known that crises lead to people reconsidering themselves in relation to both the event and wider society (Park, 2010). Likewise, it is commonly accepted that individual moral judgement in crisis situations is influenced by emotions and surrounding social beliefs and practices (Maitlis & Christianson, 2014). Participants’ narratives built around an archetypal sense of ‘good’ and ‘bad’ which infused processes of sensemaking. While the change in moral alertness and orientation varied across participants, emotions and intuitions played a significant part in the development of moral judgements, which were retrospectively reshaped into moral reasoning as a guide for action (Haidt, 2008). The polarisation of moral responses could be seen as a desire to bring order to the situation and uphold an ‘order of worth’ in which the key values of the NHS were pivotal. The narratives in the diary entries demonstrated how these values were intensely integrated into how individuals made sense of their experiences. Through moral (re)orientations, staff aimed to build on the consistency of the foundational values of the NHS, which contrasted with the constant change and confusion brought about by the pandemic and endless changes in rules and regulations. Actions in support of NHS values were seen particularly favourably, and those that contravened or did not live up to such ideals were particularly condemned. The support for this moral stance provided individuals with (self) consistency in a constantly shifting environment, offering some respite from the foil of otherwise thoroughgoing uncertainty that surrounded them (Doherty & Saunders, 2013).

With the moral sensemaking process reflected discursively in the participants’ narratives, it was possible to observe some of the mechanisms that underpinned this process. Many participants incorporated ‘othering’ within their entries, where they emphasised the moral difference between their conduct and that of others. The carving of the contrast between I and ‘other’, reinforced the position of the author of the diary as morally favourable (Turner, 2017) to the reader and themselves. Categorisation is central to sensemaking processes in chaotic situations (Weick et al., 2005). Yet, the polarisation of good and bad behaviour can lead to the formation of new social boundaries as most individuals make moral distinctions between ‘I’ and ‘others’, and attempt to situate themselves within morally good categories as part of their sensemaking processing (Barton, 2007; Copes, 2016). Such social boundaries can be divisive (Copes, 2016), negatively affecting working relationships and connections within the organisation. This could potentially lead to wider and longer‐term impacts on the quality of care given to patients, staff absenteeism, the meeting of care targets and have a knock‐on financial costs associated with these concerns (Dixon‐Woods et al., 2014; Kline, 2019; Kline & Lewis, 2019). ‘Moral othering’ could fuel further the organisational conflicts triggered by the pandemic and contribute towards additional marginalisation for some staff (Barton, 2007; Tajfel et al., 1979; Turner, 2017). This redrafting of the moral order could go on to have longer‐term implications on NHS staff as wider stressors of the pandemic are reduced and pre‐pandemic policy and working practice return.

Although narratives were representative of the participants’ individual sensemaking process, sensemaking was not carried out in isolation but in interaction with others, and was influenced by external factors and public narratives (Doherty & Saunders, 2013; Oetting & Donnermeyer, 1998). Diary entries in this study were replete with references to comments made by other individuals or by commentary from official sources, which contributed to participants’ interpretations of themselves, others and of current events. These sources, in the form of organisational messages, public health announcements and stories or images in the media, clearly shaped the way that participants made sense of the crisis. Communication from the organisation and the wider public, notably through news and social media, is likely to have fed into the newly constructed polarised moral frameworks as the public stories and images shared were themselves highly binary, for example, the positivity of the ‘NHS Heroes’, ‘clap for the NHS’ or within the hospital ‘the staff member of the week’ award, contrasted with the demonisation of ‘COVID‐19‐deniers’ and ‘anti‐vaxxers’ and the media and social media opprobrium towards people who did not abide by the COVID‐19 guidance. These stories and images were extremely prevalent throughout the observational period and were likely to have reinforced the moral conflicts perceived by the NHS staff in the study.

### Methodological contribution: Diaries and Sensemaking

While the study is limited in terms of participant numbers, its richness comes from the detailed way in which personal diaries were written and shared with the research team. The personal diary method remains relatively untapped in sensemaking studies, which is a missed opportunity given its polyphonic, multi‐layered and reflexive nature and its ability to ground individual interpretations within their wider social context. Contrary to many NHS Trusts presented on the news throughout the observational period, this hospital saw relatively few COVID‐19 cases, so the experiences of staff are likely to be different from those of other staff in the NHS at the same time. However, sensemaking is an inevitably contextual social phenomenon, and the lessons learnt here can be applied to other NHS Trusts and staff across a wide range of roles facing different types of crises.

It is of course important to note that the ways in which this data collection arose precluded us from being in possession of any diary records written prior to the period of the pandemic’s initial unfolding, which means that any comparative judgements we venture here about the accentuated nature of moral sensemaking taking place in our study period must be treated with caution. That said, the language we were confronted with in the diaries did seem redolent of polarised moral sensibility, which is what the literature would lead us to expect in times of crisis.

### Practical implications

The findings of the project were summarised in a visual display exhibited in a public area of the Trust. The art installation, an illustrated storyboard along the theme of a garden, portrayed the challenges and coping mechanisms triggered by the pandemic and projected a future where the Trust returns to full health. While this display did present findings to the public, it only described the experiences of the staff during the 3‐month period and did not include any theoretically informed analysis. The analysis for this article was carried out after the installation had been completed.

The study, artistic presentation and its underpinning management exercise represented an act of institutional sensemaking by the Trust itself. The Trust has already incorporated knowledge gained from this study into its day‐to‐day processes, and there is now a better understanding of how radical organisational and situational changes can affect the hospital in the long term. There is now a recognition that moral sensemaking and moral othering can contribute to moral distress. Moral distress describes negative feelings that people experience when, in a given situation, they are unable to carry out an action that they perceive to be the morally correct thing to do (Campbell et al., 2016). Moral distress among medical staff is currently under scrutiny in the UK, and a recent report stated that 96.4% of doctors felt that moral distress was exacerbated by the pandemic, with a belief that it would affect future working conditions (BMA, 2021). Moral distress has also been associated with resignations, reduced job satisfaction, longer‐term psychological effects, such as depression, guilt and shame and has been shown to affect quality of care, risking the health of patients and nurses (Borhani et al., 2014; Morley et al., 2019). As such, it is essential for workplaces, particularly those within health‐care environments, to consider the long‐term consequences of the moral polarisation brought about by the COVID‐19 pandemic. These findings are specific to one Trust, but may also be of interest to other NHS Trusts, and indeed other public sector organisations that may wish to reflect on the centrality of the moral and emotional dimensions of sensemaking as part of their institutional response to and management of crises.

## CONCLUSIONS

The diary entries produced by the NHS staff in our study during the initial months of the COVID‐19 pandemic provided an effective means to identify the ways in which people who work in health‐care environments make sense of sudden and dramatic crises. The study documents how participants’ sensemaking became morally infused in the immediate aftermath of the pandemic and was underpinned by a shift towards a stronger and more strictly bifurcated sense of moral judgement, which permeated participants’ perceptions of themselves, others and the institution. In so doing, the study highlights the centrality of moral intuitions and judgement in sensemaking processes in times of unprecedented societal crises. Moral judgement, defined as a highly intuitive and emotional response to exceptional environmental cues, is not simply an individual achievement but is highly influenced by social and cultural values. This may explain why NHS staff felt compelled to draw on the ‘order of worth’ espoused by the core values of the NHS to post rationalise their emotions and construct coherent accounts in order to justify their behaviours and restore a state of equilibrium that allowed them to cope more effectively with rapidly changing rules and expectations.

While sensemaking was partly beneficial for staff in that it promoted a greater sense of camaraderie and support for others (both colleagues and patients), it also appeared to have darker consequences in terms of staff wellbeing by increasing the pressure at work, the risk of burn‐out and the development of more impermeable social boundaries across the organisation through processes of moral ‘othering’.

By focussing on how the macro and micro politics of the pandemic were played out in a specialist NHS trust at the start of the COVID‐19 pandemic, the study finds that sensemaking acted as a type of repair work for individual employees at all organisational levels who invoked polarised moral judgements to justify and rationalise a broad array of associated emergent emotions, intuitions and practices. To conclude, making sense of crisis situations is inevitably an ongoing, complex and variegated enterprise whose results can be as often discomforting as they can be reassuring.

## AUTHOR CONTRIBUTIONS

**Alice Faux‐Nightingale**: Data curation (Equal); Formal analysis (Equal); Methodology (Equal); Writing – original draft (Equal). **Mihaela Kelemen**: Data curation (Equal); Formal analysis (Equal); Methodology (Equal); Supervision (Equal); Writing – original draft (Equal); Writing – review & editing (Equal). **Simon Lilley**: Formal analysis (Equal); Writing – original draft (Equal); Writing – review & editing (Equal). **Caroline Stewart**: Conceptualization (Equal); Data curation (Equal); Funding acquisition (Equal); Methodology (Equal); Project administration (Equal); Supervision (Equal); Writing – review & editing (Equal).

## CONFLICT OF INTEREST

The authors report no conflicts of interest.

## ETHICS STATEMENT

Research data were initially collected as part of a clinical audit project at the participating Trust. A retrospective ethics application was made to IRAS and granted (ID: 286648) to allow the data to be used for research purposes.

## PATIENTS CONSENT STATEMENT

Prior to engaging with this study, all participants gave consent to participate in this project and for the use of the data for research purposes beyond the initial clinical audit.

## Data Availability

The research data of this study are not available as participants did not consent to sharing their data in this way.
